# Evolutionary trade-offs between testes size and parenting in Neotropical glassfrogs

**DOI:** 10.1098/rspb.2024.0054

**Published:** 2024-02-14

**Authors:** Anyelet Valencia-Aguilar, Eva Ringler, Stefan Lüpold, Juan M. Guayasamin, Cynthia P. A. Prado

**Affiliations:** ^1^ Pós-Graduação em Ecologia, Evolução e Biodiversidade, Instituto de Biociências, São Paulo State University (Unesp), Rio Claro, São Paulo 13506-900, Brazil; ^2^ Division of Behavioural Ecology, Institute of Ecology and Evolution, University of Bern, 3032 Bern, Switzerland; ^3^ Department of Evolutionary Biology and Environmental Studies, University of Zurich, 8057 Zurich, Switzerland; ^4^ Instituto Biósfera USFQ, Laboratorio de Biología Evolutiva, Universidad San Francisco de Quito USFQ Cumbayá, Quito, Ecuador; ^5^ Departamento de Morfologia e Fisiologia Animal, FCAV, São Paulo State University (Unesp), Jaboticabal, São Paulo 14884-900, Brazil

**Keywords:** amphibians, clutch size, clutch guarding, gonadal investment, sexual selection

## Abstract

In males, large testes size signifies high sperm production and is commonly linked to heightened sperm competition levels. It may also evolve as a response to an elevated risk of sperm depletion due to multiple mating or large clutch sizes. Conversely, weapons, mate or clutch guarding may allow individuals to monopolize mating events and preclude sperm competition, thereby reducing the selection of large testes. Herein, we examined how paternal care, sexual size dimorphism (SSD), weaponry and female fecundity are linked to testes size in glassfrogs. We found that paternal care was associated with a reduction in relative testes size, suggesting an evolutionary trade-off between testes size and parenting. Although females were slightly larger than males and species with paternal care tended to have larger clutches, there was no significant relationship between SSD, clutch size and relative testes size. These findings suggest that the evolution of testes size in glassfrogs is influenced by sperm competition risk, rather than sperm depletion risk. We infer that clutch guarding precludes the risk of fertilization by other males and consequently diminishes selective pressure for larger testes. Our study highlights the prominent role of paternal care in the evolution of testes size in species with external fertilization.

## 1. Introduction

In many animal taxa, males have evolved a diverse spectrum of morphological and behavioural adaptations to increase their reproductive success relative to other males in the population [1]. The relative investment between traits under pre- (mate acquisition) or post-mating sexual selection (competitive fertilization) depends at least in part on the relative fitness accrued during either episode of selection [2,3]. For example, when growing a larger body or weapons enhances the ability of males to monopolize access to females and copulations, their risk of sperm competition would be reduced, relaxing selection on sperm production [2,4–7]. In contrast to such a negative association between traits under pre- and post-mating sexual selection, respectively, a positive relationship is predicted when the importance of sperm competition matches or exceeds that of pre-mating sexual selection [2,4,8]. Therefore, it is critical to examine the interactions between traits across episodes and types of selection to better understand the role of mate acquisition, sperm competition and sperm depletion in the evolution of reproductive strategies.

One of the traits most consistently under positive post-mating sexual selection is relative testes size, which varies considerably throughout the animal kingdom [9]. Particularly large testes relative to body size are found in species with a polyandrous mating system. If multiple males copulate (internal fertilization) or release their sperm near the same eggs (external fertilization), sperm will compete for fertilization [9–12]. Since the relative contribution of sperm from each male is one of the primary factors influencing the outcome of this competition, selection for sperm production can be intense [13]. Besides sperm competition, increased sperm production can also evolve in response to the risk of sperm depletion [14]. Sperm depletion may occur when males are selected to transfer large ejaculates to fertilize the vast numbers of eggs released by females in externally fertilizing species [9,15,16] or to compensate for sperm loss in the female reproductive tract in internally fertilizing species [17–19]. Similarly, males may deplete their sperm reserves by mating frequently within a short period [20,21]. Increased sperm production can be metabolically costly [22–24], and males must trade these investments against others, such as body size, sexual ornaments or weaponry [3,25]. Comparative research in primates that investigated the relationship between testes size, male ornaments, sexual size dimorphism (SSD) and mating systems has provided key insights into the evolutionary trade-offs of different reproductive strategies [8,26–28]. In species that live in multi-male groups (e.g. bonobos, chimpanzees), males often produce more sperm and have larger testes relative to their body size, compared to species that live in single-male groups (e.g. gorilla, orangutan), where risks of sperm competition and sperm depletion are reduced.

While relative testes size is one indicator of the level of sperm competition, behavioural (e.g. mate guarding) or morphological adaptations (e.g. body size, weapons) may also evolve to enhance fertilization success [2,3]. Mate guarding is a common strategy in internally fertilizing species to avoid reproductive interference by conspecific male competitors [29–31]. In externally fertilizing species, behavioural strategies such as territoriality and parental care (e.g. clutch guarding) may have a similar function by ensuring a high certainty of paternity [32–34]. Indeed, male-only care in the form of clutch guarding, which has independently evolved in many animal taxa [35–37], appears to be particularly prevalent in species with external fertilization [38,39]. Variation in the level of paternal effort necessary for offspring survival and development can lead to an allocation trade-off between male parental effort and investment into gametes (e.g. [40,41]). Studies on the role of paternal care in the evolution of testes size have reported contrasting results; that is, negative covariation in some taxa (e.g. birds [20], mammals [21] or fish [42]), a positive correlation in others (e.g. cuckoos [43]) or no association (e.g. birds [44], fish [45] or anurans [34]). One possible cause of these contrasting patterns is a difference in fertilization modes between taxa. For example, in internally fertilizing species, a negative relationship between male parental care and testes size likely indicates that parenting reduces the reproductive rate of the carer, resulting in less intense selection on sperm production. In external fertilizers, this relationship probably reflects a trade-off between pre- and post-mating sexual selection. These taxon-specific patterns also suggest that the link between paternal care and testes size is more complex than previously thought, with variation in mating systems, reproductive tactics or investment in other traits (e.g. sexual ornaments or armaments) contributing to the evolution of testes size in vertebrates (see also [46]).

Despite recent theory suggesting that males investing in care will allocate fewer resources to ejaculate production [46,47], the empirical evidence for such a relationship remains scarce [47]. Hence, we studied how male parental care, SSD, weaponry and clutch size are linked to testes size evolution, focusing on the macroevolutionary patterns among these traits across glassfrogs (Centrolenidae), a Neotropical frog family that exhibits variation in all these traits [48–50].

Centrolenid frogs are nocturnal and breed along streams where females lay eggs on the upper or underside of leaves hanging above water while the males, which are slightly smaller in size, release sperm to fertilize the eggs [50]. The mating system is sequentially promiscuous, and males of several species show paternal care by attending eggs, particularly in *Hyalinobatrachium*, one of the major clades [50,51]. In addition, males of several glassfrog species (e.g. *Centrolene*, *Chimerella*, *Espadarana*, *Nymphargus*, *Sachatamia*) have prominent humeral spines, bony protrusions from their upper arm bones that they use in fights with other males (see [50,52]). Here, we tested the hypothesis that male glassfrogs differentially invest in traits under pre- or post-mating sexual selection in response to interspecific variation in clutch sizes and the occurrence of paternal care. Specifically, we predicted that traits like male-biased SSD, humeral spines and clutch guarding would be associated with a low sperm competition risk, resulting in relatively small testes due to relaxed selection on fertilization effort. We further expected that if fertilization success is affected by sperm depletion, males of species with large egg clutches would have evolved relatively larger testes. Finally, to the extent that male-biased SSD and humeral spines are associated with pre-mating competition, we predicted that species with relatively large males should be more likely to express humeral spines.

## 2. Material and methods

### (a) Data collection

**Figure 1.**
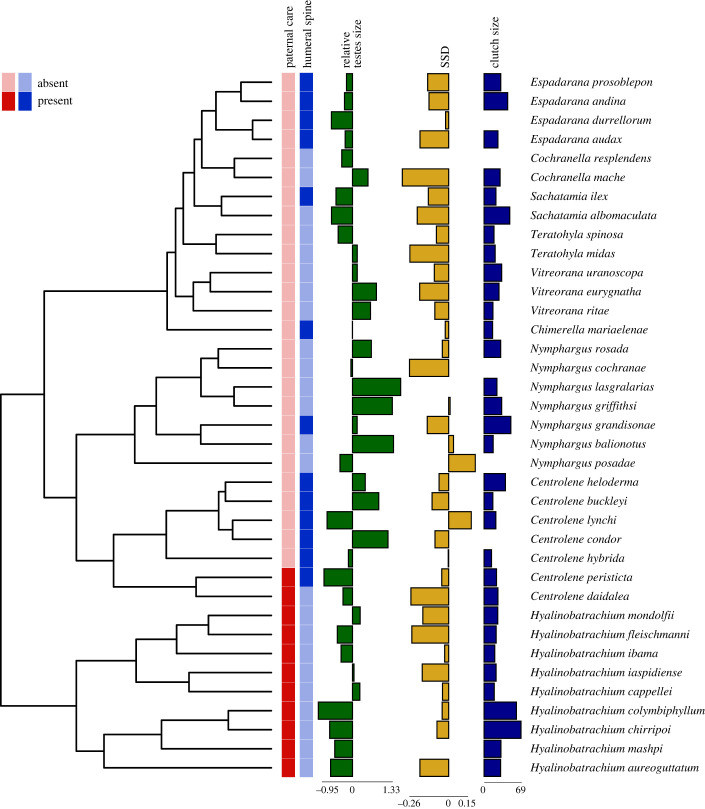
Reconstructed phylogeny of the glassfrog species examined in our study. The tips of the phylogeny are annotated with five traits: the presence or absence of paternal care and humeral spines, relative testes size (represented as residuals from a log-log regression against male snout-vent length, SVL), sexual size dimorphism (SSD, calculated as the log-ratio of male SVL to female SVL) and clutch size.

We collected reproductive data during fieldwork in Brazil (Fazenda São Nicolau 9°49′10.1″ S, 58°15′29.9″W, 2016 and 2017) and Ecuador (Itapoa 0°07'22.2″ N, 79°16'16.2″ W and Canandé 0°31'18.6″ N, 79°08'09.8″ W reserves, 2018 and 2019), as well as from specimens deposited in the Zoology Museum at the Pontificia Universidad Católica del Ecuador (QCAZ), Instituto Nacional de Biodiversidad (INABIO, Ecuador), Centro Jambatu de Investigación y Conservación de Anfibios (Ecuador), the Célio F.B. Haddad (CFBH) Amphibian Collection, Universidade Estadual Paulista, Rio Claro, São Paulo (Brazil), the Coleção de Anfíbios do Centro de Coleções Taxonômicas da Universidade Federal de Minas Gerais (UFMG, Brazil) and material provided by Dr Marco Rada from Colombia. We supplemented the dataset with data about the body size and clutch size of females from the primary literature, an online database [53], and peer-reviewed books [48,54]. Our final dataset (electronic supplementary material, dataset S1; figure 1) included complete male data for 37 (23%) of the 160 described glassfrog species (Centrolenidae), distributed across nine of the 12 glassfrog genera [50,52,55]. We sampled nine species of the Hyalinobatrachinae subfamily, which consists of 37 species across two genera [50]. All our sampled species of this subfamily came from the *Hyalinobatrachium* genus and are known to exhibit paternal care [51]. The remaining 28 studied species were spread across eight of the nine described genera of the Centroleninae subfamily, including seven each of *Centrolene* and *Nymphargus*, four of *Espadarana*, three of *Vitreorana,* two each of *Cochranella*, *Sachatamia* and *Teratohyla* and one species of *Chimerella*. Across the 121 species of this subfamily [50,55], paternal care is known to occur in only five of the 25 *Centrolene* species [51], two of which were included in our dataset.

### (b) Life-history variables

For the 37 species of glassfrogs, we collected information about the following variables: (1) male snout–vent length (SVL), (2) female SVL, (3) clutch size, (4) testes size (volume), (5) humeral spine and (6) paternal care. Both humeral spine and paternal care were treated as binary variables (present or absent). SVL was measured for males and females with digital callipers to the nearest 0.1 mm. Clutch size was defined as the average number of eggs laid during one spawning event or the number of mature oocytes in preserved specimens. Testes size of each species was calculated as 4/3π × *a*^2^ × *b*, where *a* and *b* are the average width and length of the right testis per species, respectively [56], measured using an ocular micrometre (0.1 mm scale) fitted to a stereomicroscope (Zeiss Stemi SV11). We measured testes sizes in 37 glassfrog species collected for some of the co-authors during fieldwork or provided by collections in Ecuador and Brazil (electronic supplementary material, dataset S1). Fieldwork was conducted during the local rainy season from 2015 to 2017 in Brazil and from 2018 to 2019 in Ecuador. In some anuran species, testes tend to regress outside the breeding season [57,58] or vary in size depending on environmental conditions [59] or male–male interactions [45,60]. Hence, for each species, we measured only specimens that had been collected within their respective breeding season in the same locality and period. Because of these constraints, we were able to measure only up to seven males per species (except for *H. aureoguttatum* with 15 males: electronic supplementary material, dataset S1).

### (c) Phylogenetic inference

For our trait evolution analyses, we used Guayasamin *et al.*'s [50] phylogeny. The topology was inferred using a Bayesian analysis and divergence dating method implemented in BEAST v.2.4.5 (for a detailed description see [50]). Briefly, the analysed dataset contains complete or partial sequences of 10 genes for 113 named species, and 24 putative new species from all 12 glassfrog genera, with an outgroup of 49 taxa from a large range of families including all three species of Allophrynidae, the sister group to the Centrolenidae [52]. Evolutionary models and partitions are detailed in [50]. The resulting phylogeny is the result of Markov chain Monte Carlo searches for a total of 100 million generations, sampling every 10 000 generations. Stationarity was assessed by examining the standard deviation of the split frequencies and by plotting the log-likelihood per generation, using Tracer v.1.5; trees generated before stationarity were discarded as ‘burn-in’, which was 20% of trees.

### (d) Phylogenetic comparative analysis

We performed all analyses in R v.4.2.1 [61], with all continuous variables log-transformed before inclusion into models. Since traits can covary between species due to their common ancestry, we accounted for phylogenetic relationships based on the time-calibrated phylogeny from Guayasamin *et al.* [50]. We conducted phylogenetic generalized least-squares (PGLS) or phylogenetic logistic regressions as implemented in the R package phylolm [62], determining the 95% confidence intervals for both the model estimates and the phylogenetic scaling parameter *λ* (in linear models) or *α* (in logistic models) by bootstrapping across 100 fitted replicates. To address intraspecific variation and phylogenetic uncertainty (both topology and branch lengths) in more detail, we further resampled (with replacement) averaged individual measures within species and took individual trees from our posterior tree sample generated during Bayesian phylogenetic reconstruction. Across 1000 resampled datasets and trees, we repeated all analyses and found that the estimated coefficients generally were robust despite the relatively small sample sizes within species (electronic supplementary material, figures S1–S10).

## 3. Results

A bootstrapped PGLS model, accounting for intraspecific variation and phylogenetic uncertainty confirmed a significant correlation between testes size and male SVL (*n* = 37 species; *β* = 3.40 [95%CI: 2.20, 4.50], *t*_35_ = 5.68, *p* < 0.001, *λ* = 0.52 [95%CI: < 0.01, 1.00]). The 95%CI of this allometric slope between testes volume and SVL as a linear measure of body size, included three, indicating that testes size scaled proportionately (isometrically) with body size across our sample of species.

**Figure 2.**
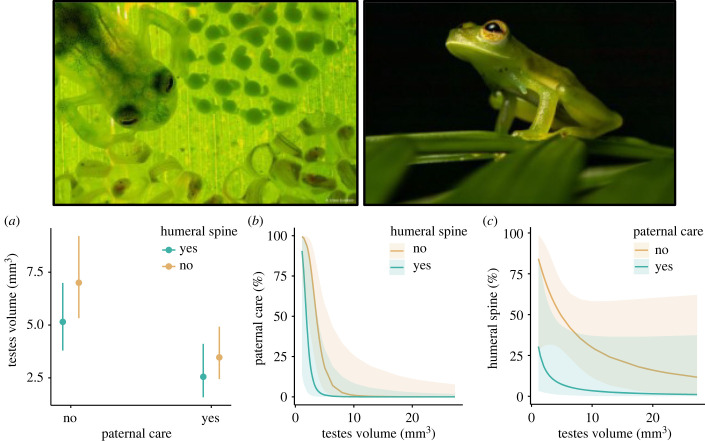
Relationships of (*a*) testis volume (back-transformed from log scale), (*b*) the presence/absence of paternal care, and (*c*) the presence/absence of humeral spines as response variables with the other two traits and log SVL as predictors. Top left, male of *Hyalinobatrachium valerioi* caring for clutches (Photo: Jaime Culebras). Top right, male of *Espadarana prosoblepon* with the humeral spine (Photo: Francesca Angiolani).

Next, we tested the links between relative testes size, the presence or absence of paternal care and the presence or absence of humeral spines across *n* = 37 species with complete data. Accounting for body size (*β* = 3.15 [2.17, 4.23], *t*_33_ = 5.42, *p* < 0.001) and phylogeny (*λ* = 0.33 [less than 0.01, 0.91]), males had relatively larger testes in species with no paternal care (*β* = −0.80 [−1.32, −0.30], *t*_33_ = −2.88, *p* = 0.007), while the presence of humeral spines had no statistically significant effect despite a weak negative bias in the bootstrap interval (*β* = −0.30 [−0.66, 0.08], *t*_33_ = −1.53, *p* = 0.13; figure 2*a*). In a logistic regression across the same species, controlling for SVL (*β* = 1.20 [0.51, 3.58], *z* = 0.42, *p* = 0.68) and phylogeny (*α* = 1.66 [0.24, 719.56]), paternal care was found significantly more often in species with relatively small testes (*β* = −1.58 [−4.26, −0.70], *z* = −2.23, *p* = 0.03; figure 2*b*), while a significant effect was detected for the presence of humeral spines (*β* = −0.86 [−3.57, 0.53], *z* = −1.31, *p* = 0.19). Finally, again controlling for SVL (*β* = 7.19 [5.95, 9.11], *z* = 1.86, *p* = 0.06) and phylogeny (*α* = 13.83 [0.24, 722.49]), the bootstrap interval of testes size as a predictor of the presence of humeral spines was biased towards negative values, but not statistically significantly (*β* = −1.28 [−2.57, −0.20], *z* = −1.53, *p* = 0.12). No such trend was found for the occurrence of paternal care (*β* = −1.78 [−5.94, 0.68], *z* = −1.30, *p* = 0.20; figure 2*c*).

Each of the three traits could further be linked, in its own way, to the degree of pre-mating sexual selection (see §1). Whereas the relationship between male-biased SSD and testes size shifts from positive to negative across taxa with an increasing prevalence of female monopolization [2], paternal care and the expression of sexual weaponry have been positively associated within some taxa (e.g. anurans [63], fishes [64], insects [65]). Hence, we also tested if testes size, paternal care and humeral spine in glassfrogs were associated with SSD, another trait often responding to pre-mating sexual selection. Across 34 species with complete data, we found no evidence that SSD, measured as log(male SVL / female SVL) and controlling for male SVL, was significantly related to relative testes size (SSD: *β* = −1.16 [−3.13, 0.81], *t*_31_ = −1.08, *p* = 0.29; SVL: *β* = 3.81 [2.58, 5.02], *t*_31_ = 5.67, *p* < 0.001; *λ* = 0.47 [0.01, 0.99]). Across the same species, we also found no evidence for an effect of SSD on paternal care (SSD: *β* = 0.57 [−4.38, 4.58], *z* = 0.16, *p* = 0.88; SVL: *β* = −6.21 [−8.11, −5.69], *z* = −2.22, *p* = 0.03; *α* = 4.31 [0.25, 722.47]) nor on humeral spines (SSD: *β* = 1.11 [−4.11, 5.20], *z* = 0.24, *p* = 0.81; SVL: *β* = 5.18 [4.39, 6.76], *z* = 1.76, *P* = 0.08; *α* = 16.06 [0.25, 724.87]).

Finally, due to paternal care could be a response to female clutch investments, we further examined relationships between relative clutch size and paternal care across *n* = 30 species with complete data. Here, controlling for female SVL (*β* = 1.70 [0.85, 2.54], *t*_27_ = 3.77, *p* < 0.001), species with paternal care did not lay larger clutches than those without paternal care (*β* = 0.25 [−0.07, 0.56], *t*_27_ = 1.53, *p* = 0.14, *λ* = 0.16 [less than 0.01, 0.84]). However, paternal care tended to be more probable in species where females lay relatively large clutches (paternal care: *β* = −2.83 [0.64, 5.30], *z* = −1.91, *p* = 0.06, female SVL: *β* = −11.77 [−14.16, −9.26], *z* = −2.18, *p* = 0.03; *α* = 5.29 [0.24, 713.26]). Similar to paternal care, clutch size could also affect relative testes size, for example via selection on sperm production, in response to the risk of sperm depletion by fertilizing large clutches [66]. However, we found no evidence for a response of relative testes size to clutch size variation (clutch size: *β* = −0.37 [−0.88, 0.13], *t*_27_ = −1.41, *p* = 0.17; SVL: *β* = 4.29 [2.73, 5.86], *t*_27_ = 5.26, *p* < 0.001; *λ* = 0.67 [less than 0.01, 1.00]).

## 4. Discussion

Our results suggest that glassfrogs may evolutionarily trade-off relative testes size with paternal care. A similar pattern regarding the presence of humeral spines cannot be rejected but received less support in our dataset. Moreover, species with relatively large clutches were more likely to show paternal care than those with smaller clutches, but there was no significant relationship between clutch size and relative testes size.

Sperm competition plays an important role in the evolution of paternal care, as the likelihood of males investing in parental care generally declines with a decrease in their probability of paternity [44,47]. Previous comparative studies of anurans have shown that high levels of sperm competition are also likely to be the main factor selecting for larger testes or ejaculates, while males of species with hidden nests (i.e. eggs not exposed to sneakers) and a low sperm competition risk have relatively smaller testes [6,15,67,68]. In this context, our results suggest that paternal care may lower the sperm competition risk via clutch guarding. In anurans, sperm may migrate through the gelatinous clutch matrix for an extended period (see [69]), favouring the post-mating clutch piracy in some species, where sneaker males fertilize remaining oocytes immediately after oviposition [70]. Thus, by protecting their clutches from potential sneakers, glassfrog males may reduce the need to invest in sperm production, ultimately decreasing testes size or limiting its evolutionary increase. Similarly, gladiator frogs in the *Boana faber* group that aggressively defend constructed nests against intruder males, and exhibit short-term paternal care, also have smaller testes compared to closely related species [67]. Although few studies have assessed paternity in glassfrogs, to date there is no evidence of sperm competition or multiple paternity in species that provide paternal care (*Hyalinobatrachium valerioi* [71], *H. cappellei* [72]). Thus, further studies comparing the paternity in clutches of glassfrog species with and without parental care might help us to understand whether the emergence of paternal care is linked to the evolution of small testes size via a reduction in sperm competition risk.

In general, the likelihood of paternal care evolving is expected to increase with the certainty of paternity [47]. Different forms of parental care vary in their temporal and energetic costs to the carer, and more costly forms might constrain male investment in fertilization effort and consequently in testes size. Clutch guarding, for example, might constrain the number of females a male can mate with during the care period. In the genera *Hyalinobatrachium* and *Centrolene*, attending males simultaneously care for up to six and two clutches (from different females), respectively, for several weeks [71,73–75]. Hence, males that do not invest in clutch guarding (e.g. *Espadarana*, *Nymphargus*, *Sachatamia*, *Teratohyla*, *Vitreorana*) might allocate more resources to mating, including testes size.

Besides sperm production, testes size has also been associated with testosterone levels, which play a central role in the expression of numerous sexual traits and behaviours [76–80]. However, although levels of circulating testosterone are often positively correlated with male testes size and aggressive behaviours [16,78], elevated androgen levels might also interfere with male parental care [79–83]. In some fish, anuran and bird species, male testosterone levels are typically high during the mating period but decrease when males start to care for their offspring [79,83], suggesting a negative effect of testosterone on care provisioning. In glassfrogs, male aggressive behaviours are more frequently observed in species without paternal care [84,85], which on average have relatively large testes (this study). In glassfrogs, it is thus possible that when the fitness benefits of clutch guarding may surpass those of male aggression, males will have lower levels of testosterone, decreasing both testes size and male aggressiveness. Further investigation of the hormonal mechanisms underlying parental behaviour in these frogs might help us to gain new insights into the ultimate cause of testes size variation and its influence on aggressiveness and parental care.

Another possibility is that relatively large testes in glassfrogs without paternal care might be associated with a higher male mating effort. In general, males that do not provide care are expected to fertilize more egg clutches during the breeding season than their caring counterparts [86]. With many fertilization events over a short period, selection should favour relatively larger testes to maximize sperm production and minimize the risk of (temporary) sperm depletion [87]. Males of glassfrog species, such as *Teratohyla spinosa*, *Sachatamia albomaculata* and *Vitreorana uranoscopa,* do not provide parental care and can mate with more than one female in the same night (A.V.-A. 2023, personal observation). Hence, differences in sperm production across species are possibly related to variations in resource allocation between mating and parenting [36]. Paternal effort could lead to a decrease in gonadal investment because if a male invests more in clutch guarding, he will have less energy to invest in survival, growth, or gamete production [88]. In fact, sperm production can be costly [89–91], and because organisms have limited energy to invest in reproduction, it is expected that the allocation of resources towards either mating or parenting will be optimized to maximize male fitness [47].

Parental care is generally associated with large eggs and small clutches/littres (e.g. anurans [92–94], fish [95], mammals [96]). However, we found a trend towards larger clutches in glassfrogs with paternal care. A possible explanation is that clutch size actually reflects the indirect effect of another clutch trait on paternal care, such as egg-clutch jelly, because we also found no significant differences in clutch size between species with and without paternal care [49]. Females of the species without paternal care (e.g. genera *Centrolene*, *Chimerella*, *Cochranella*, *Espadarana*, *Nymphargus*, *Sachatamia*, *Teratohyla* and *Vitreorana*) lay an average of 14–50 eggs per clutch (electronic supplementary material, dataset S1), while clutch sizes in species with paternal care (*Hyalinobatrachium* and *Centrolene*) range between 19 and 69 eggs (electronic supplementary material, dataset S1). Egg-clutch jelly, on the other hand, is quite diverse among glassfrog species and critical for embryo survival [49]. Clutches differ in the maternal jelly products surrounding the vitelline membranes, ranging from simple clumps of eggs with only thin jelly capsules, to eggs embedded in large gelatinous structures [49]. Species with paternal care tend to have clutches with a simple clump of egg (flat layer of eggs touching each other without jelly exposed between them), deposited on the underside of the leaves. In species without paternal care, clutches range from simple clumps of eggs to eggs embedded in a rich jelly matrix, which can be found in a diversity of arboreal and terrestrial substrates (e.g. upper surfaces of leaves, on rocks in spray zones, moss on branches) [49,50]. Jelly contributions might determine the potential of a clutch to absorb and store water, increasing embryo survival [49,51]. Indeed, the survival of clutches without a jelly-rich matrix around eggs tends to depend on dehydration prevention by parental care (as observed in species of *Hyalinobatrachium* and some *Centrolene*). Conversely, clutches with a jelly-rich matrix hydrate faster and survive longer without rehydration as observed in species without paternal care in the genera *Chimerella*, *Cochranella*, *Espadarana*, *Nymphargus*, *Sachatamia*, *Teratohyla* and *Vitreorana*, [49]. Therefore, we suggest that, like egg and clutch size, the egg-clutch jelly structure could also be associated with parental care in glassfrogs. This hypothesis is yet to be tested. Incorporating different lineage-specific life-history traits in further comparative analyses might uncover interesting relationships that so far have been masked or not considered.

SSD in glassfrogs was predominantly biased towards females (30 out of 34 species), corroborating earlier SSD studies in anurans [63,97], but it was not related to relative testes size, paternal care, or humeral spines expression. Han & Fu [63] found that a decrease in female-biased SSD towards monomorphism or male-biased SSD was associated with the evolution of parental care in anurans. However, later studies in anurans did not support this result [97] (this study). Male-biased SSD is generally observed in species with high levels of territoriality or sperm competition, or when providing care allows males to increase mating opportunities (e.g. fish [98,99]). Although in glassfrogs, caring males mate more frequently than non-caring males [72], we found males of caring species to be smaller than those of non-caring species (electronic supplementary material, dataset S1), probably because of the energetic costs of caring [32,40]. Additionally, male-biased SSD has also been correlated with the presence of male combat behaviour, the expression of weapons (e.g. spines and tusks), in some anuran groups [100] and testes size across diverse taxa [2]. Although our data do not support these findings, new systematic data on testes and weapons size of a greater number of frog species could help elucidate the type and direction of a relationship between SSD and the expression of other sexually selected traits.

Relatively larger testes may also be favoured by selection in species with large clutches because these require more sperm to be fertilized [16,92]. A comparative study in anurans showed a positive correlation between testes size and clutch size [16]. However, subsequent studies across more than 180 Australian anurans found no such association, but instead a positive correlation between relative testes size and sperm competition risk linked to different oviposition sites [58,93]. Our results also showed no association between testes size and clutch size. In fact, clutch size did not differ significantly between species with and without paternal care, suggesting that egg number is unlikely to be one of the main factors driving testes size evolution in glassfrogs. Besides clutch size, the type of clutch (jelly masses or foam nests), mating system and spawning location (e.g. aquatic versus terrestrial, hidden versus exposed) are other factors that have previously been reported to influence ejaculate expenditure, and thus testes size in anurans [15,67,68,94]. It seems likely that variation in egg-clutch structure (e.g. simple or jelly-rich) and oviposition sites (e.g. sheltered or exposed to rain) might also affect ejaculate expenditure and testes size in glassfrogs [49,50]. Indeed, in species without paternal care, clutches are jelly-rich and laid in sites exposed to rain [49], which could increase the risk of sperm loss and so favour larger testes and higher sperm production to ensure clutch fertilization.

Finally, our analyses suggest a negative association between testicular investment and the presence of humeral spines across glassfrogs, even though the evidence for such a trend was at best weak and somewhat ambiguous between bootstraps and traditional frequentist statistics. It is possible that quantitative data on these spines would provide a clearer picture than their mere presence/absence, but such data were not generally available. If true, however, these results would further point towards evolutionary trade-offs between different male allocation strategies to maximize their fitness in different mating contexts. For example, when males are not constrained by attending egg clutches, they can invest more in mating, possibly resulting in more intense male–male competition for females and the fertilization of clutches. To the extent that the presence of humeral spines reflects more intense pre-mating sexual selection, an associated reduction in relative testes size could be the result of relaxed post-mating sexual selection because success in male-male contests yields relatively greater marginal fitness benefits than sperm competitiveness [4]. More work is clearly needed to elucidate a possible link between humeral spines and other costly traits for a more complete understanding of male allocation patterns.

In conclusion, our results demonstrate the importance of paternal care in the evolution of testes size in species with external fertilization. In addition, although the evolutionary associations between weapons and testes tend to covary positively in taxa without or low levels of female monopolization [25], we found a negative relationship between those traits. Our results reveal important links between sexual and non-sexual selection as well as between pre- and pos-tmating sexual selection that jointly shape the evolution of testes size in glassfrogs, opening an avenue for investigation in other animal groups.

## Data Availability

All data generated or analysed during this study are included in this published article. Supplementary material is available online [101].
